# Social adversity during juvenile age but not adulthood increases susceptibility to an immune challenge later in life

**DOI:** 10.1016/j.ynstr.2023.100526

**Published:** 2023-02-08

**Authors:** Cyprien G.J. Guerrin, Janine Doorduin, Kavya Prasad, Daniel A. Vazquez-Matias, Lara Barazzuol, Erik F.J. de Vries

**Affiliations:** a Department of Nuclear Medicine and Molecular Imaging, University Medical Center Groningen, University of Groningen, Hanzeplein 1, 9713, GZ, Groningen, the Netherlands; b Department of Radiation Oncology, University of Groningen, University Medical Center Groningen, Hanzeplein 1, 9713 GZ, Groningen, the Netherlands; c Department of Biomedical Sciences of Cells and Systems, University of Groningen, University Medical Center Groningen, Hanzeplein 1, 9713 GZ, Groningen, the Netherlands

**Keywords:** Social stress, Psychopathologies, Microglia priming, Dual hit model, Adolescent social defeat, LPS challenge

## Abstract

Adverse experiences in early life can increase mental vulnerability to immune challenges experienced later in life, which may induce the development of stress-related psychopathologies. Here, we investigated whether the combined effect of both events is higher if the first adverse experience occurs when the brain is still in development. Therefore, male Wistar rats were exposed to repeated social defeat (RSD, first hit) during juvenile age or adulthood and to an immune challenge consisting of a single injection of lipopolysaccharide (LPS, second hit) in adulthood. Control animals were not exposed to RSD, but only to the LPS challenge. Translocator protein density, a marker for reactive microglia, microglia cell density and plasma corticosterone levels were measured using in vivo [^11^C]PBR28 positron emission tomography, iba1 immunostaining, and corticosterone ELISA, respectively. Anhedonia, social behavior and anxiety were measured with the sucrose preference, social interaction, and open field tests, respectively. Rats exposed to RSD during juvenile age exhibited enhanced anhedonia and social interaction dysfunction after an immune challenge in adulthood. This enhanced susceptibility was not observed in rats exposed to RSD during adulthood. In addition, exposure to RSD synergistically increased microglia cell density and glial reactivity to the LPS challenge. This increase in microglia cell density and reactivity to the LPS challenge was more pronounced in rats exposed to RSD during juvenile age than in adulthood. Exposure to RSD alone in juvenile age or adulthood induced similar short-term anhedonia, a long-lasting increase in plasma corticosterone and microglial activity, but no change in anxiety and social behavior. Our findings indicate that exposure to social stress during juvenile age, but not adulthood, primes the immune system and increases the sensitivity to an immune challenge experienced later in life. This suggests that juvenile social stress can have more deleterious effects in the long term than similar stress in adulthood.

## Highlights

• Juvenile social stress induces a long-term increase in microglia reactivity.
• Social stress induces a long-term increase in plasma corticosterone levels.
• Juvenile social stress increases susceptibility to an immune challenge.
• Social stress primes microglia and alters its later response to infection.
• LPS challenge and social stress synergistically induce anhedonia and social alteration.

## Graphical abstract

**Figure:**
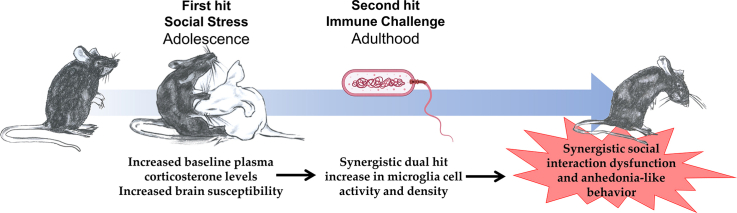

## 1 Introduction

Traumatic social events can place individuals at risk for developing stress-related psychopathologies and psychiatric disorders later in life (Varchmin et al., 2021; Varese et al., 2012). Psychological trauma, despite its relatively frequent occurrence (Comijs et al., 2007; Dong et al., 2004), is usually not sufficient for disease induction (Stilo and Murray, 2010) and seems to have a rather modest effect in large populations (Varese et al., 2012). To explain this modest effect, it has been proposed that social adversity can render an individual more vulnerable to the pathological effects of a second postnatal stimulus, such as infection or sepsis (Bayer et al., 1999; Guerrin et al., 2021; Hjorthøj et al., 2020).

Social adversity activates the hypothalamus-pituitary-adrenal axis (HPA) and can chronically increase glucocorticoids levels. A higher level of glucocorticoids is an indicator of increased stress sensitivity, which is commonly observed in individuals exposed to childhood adversity and at risk of psychosis (Reininghaus et al., 2016; Walder et al., 2000). Furthermore, higher glucocorticoid level is an important physiological process in the development of psychotic experiences in the early stages of schizophrenia (Reininghaus et al., 2016; Walder et al., 2000). Increased levels of glucocorticoids play a complex modulating role on the immune system by having anti-and pro-inflammatory properties (Yeager et al., 2011). Higher number of reactive microglia and other neuroinflammatory changes, including higher brain levels of pro-inflammatory cytokines, are often observed in patients with neurodevelopmental disorders, such as schizophrenia and autism, or mood disorders, such as bipolar disorders and depression (Hughes and Ashwood, 2020; Meltzer and Van de Water, 2017). Altogether, the stress associated with social adversity could dysregulate the HPA axis and prime the immune system to an aberrant response to a second postnatal stimulus. Yet, vulnerability to social adversity seems to depend on age. Social trauma experienced during juvenile age, a critical phase during which the brain undergoes many neurodevelopmental changes, is believed to have more detrimental effects than trauma experienced in adulthood when the brain is fully developed (Varchmin et al., 2021). However, this hypothesis still awaits direct verification.

In this study, we tested whether exposure to social adversity during juvenile age or adulthood affects glial reactivity, glucocorticoid levels, and behavior differently and whether such adversity alters the response to infection later in life. To achieve social adversity, repeated social defeat (RSD) was performed in juvenile and adult rats. Rats exposed to RSD were previously found to display altered behavioral phenotypes relevant to stress-related psychopathologies, including increased anxiety, social withdrawal, and anhedonia (Iñiguez et al., 2014; Vidal et al., 2007). Infection later in life was performed by injecting lipopolysaccharide (LPS), the major component of the outer membrane of gram-negative bacteria, a factor shown to induce sickness behavior in humans and rodents (Bassi et al., 2012; Fullerton et al., 2016). To measure glial reactivity to the stressors and serum glucocorticoid levels, we performed non-invasive Positron Emission Tomography (PET) imaging using the PET tracer [^11^C]PBR28 to measure translocator protein (TSPO) levels, protein associated with reactive microglia, and took blood samples, at different time points. PET imaging allows to measure TSPO levels in vivo non-invasively and, therefore, to conduct longitudinal within-group comparisons. Additionally, it offers translational value as TSPO tracers are also used and validated clinically to measure neuroinflammation. Potential differences in microglia density were examined by quantification of cells immunoreactive for the ionized calcium-binding adaptor molecule 1 (Iba1) on day 93. To measure anxiety-like behavior, social behavior, and anhedonia, symptoms often observed in psychopathologies, we longitudinally performed the open field test (OFT), social interaction test (SIT), and sucrose preference test (SPT), respectively.

## 2 Materials and methods

### 2.1 Animals

All experiments were performed in accordance with European Directive 2010/63/EU and the Law on Animal experiments in the Netherlands. Twenty-four male outbred Wistar Unilever rats (HsdCpd:WU, age 21–23 days) were purchased from Envigo (Horst, The Netherlands), housed in pairs with food and water available *ad libitum.* Rats were allowed to acclimatize for seven days before the start of the experiments. Housing rooms were humidity-controlled and thermo-regulated (21±2 °C), with a 12:12-h light:dark cycle (lights on at 7 a.m.). Day 35–39 and day 63–67 were considered as juvenile age and adulthood, respectively (Babikian et al., 2010; Semple et al., 2013). Only males were included in this study because the RSD model of social stress is well validated in males but not females due to the males innate territorial aggression toward other males intruding their territory, behavior rarely observed in female rats.

### 2.2 Experimental design

**Fig. 1:**
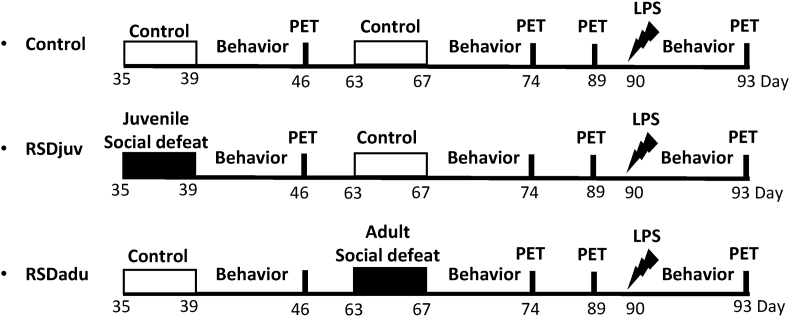
**Study design.** Behavior consists of sucrose preference test (day 40, 62, 68, 89 and 91), open field and social interaction tests (day 40,74, and 91). Blood samples were collected before each PET scan. Rats were sacrificed following the last PET scan on day 93.

Male rats were randomly divided into three groups: (1) rats not exposed to social defeat (control, n = 8), (2) rats exposed to social defeat at a juvenile age (day 35–39, RSDjuv, n = 8) but not adulthood, and (3) rats exposed to social defeat during adulthood (day 63–67, RSDadu, n = 8) but not juvenile age. All animals were injected with LPS on day 90. PET scans to measure glial activity, blood samples to measure plasma corticosterone and behavioral tests to measure anhedonia, anxiety and sociability were performed as shown in Fig. 1.

### 2.3 Repeated social defeat

12–16 week old male Long Evans rats (HsdBlu:LE – Envigo, USA) were used as residents. To encourage territoriality, each male resident was housed in a large cage (80*50*40 cm) with an ovariectomized female Long Evans rat that underwent tubal ligation for at least one week before the screening. The screening consisted of measuring attack latency and submission time in RSD sessions on five consecutive days to select rats displaying the desired aggressive behavior and exclude rats showing signs of violence (attack latency <10 s and attempts to kill the intruder by attacking vital zones), or non-aggressive behavior (attack latency>60s, absence of submission or submission time >120s, or the resident being submitted) (Moraga-Amaro et al., 2022).

The RSD protocol was performed between 1 and 4 p.m. Female Long Evans rats were removed from the resident cages 1 h before the exposure of the experimental rat. The session lasted 1 h and started with the introduction of the experimental rat in the cage of the resident, after which they were allowed to interact until the experimental rat showed a submissive posture for at least 5 s or after 10 min of interaction. Subsequently, the experimental rat was placed inside a wire mesh cage and put back in the cage of the resident to allow for visual, auditory, and olfactory interactions for the remainder of the 1 h. The experimental rat was exposed to five different residents for five consecutive days. Control rats were placed in a wire mesh cage inside a clean cage for a duration of 1 h.

RSDjuv and RSDadu rats were individually housed during the RSD protocol and the period thereafter during which the behavioral tests and PET scan took place, i.e. on day 35–46 and day 63–74, respectively, as social interaction during group housing can counteract the effect of social defeat. The control group remained housed in pairs during the RSD protocols. Hereafter the rats were group housed again.

### 2.4 LPS administration

On day 90, all the rats were intraperitoneally injected with a solution of 1 mL of 2 mg/kg of *E. coli* lipopolysaccharide (Sigma Aldrich, L2630) dissolved in Dulbecco's phosphate-buffered saline (PBS).

### 2.5 Sucrose preference test

Sucrose preference tests were performed on days 34, 40, 62, 68, 89, and 91 to measure anhedonia. SPT training consisted of placing a bottle with 1% sucrose in water in the cage for 1 h on four consecutive days (day 30–33), followed by overnight exposure to two bottles, one filled with 1% sucrose solution and the other with normal drinking water. The SPT consisted of overnight exposure to two bottles, one filled with a 1% sucrose solution, and one filled with drinking water. The sucrose preference was measured as the outcome parameter and was calculated according to the following formula: sucrose preference (%) = [Sucrose intake (mL)/(Sucrose intake (mL)+ Water intake (mL))] *100%.

### 2.6 Open field test

Open field tests (OFT) were performed on day 40, 68 and 91 to measure anxiety-like behavior and motility. Rats were allowed to acclimatize to the experimental room for 1 h before being placed in a circular area (100 cm diameter) for 5 min. Time spent in the center (>10 cm from the wall) was measured to determine the level of anxiety-like behavior. Behavior was video recorded and analyzed offline using Ethovision XT14 (Noldus Information Technology, Wageningen, The Netherlands). A 10% ethanol solution was used to clean the arena after each session.

### 2.7 Social interaction test

Social interaction tests (SIT) were performed on day 40, 68 and 91 to measure social behavior. The test was performed following the OFT. To account for the size differences and to allow for closer interactions between the rats, the first SIT was performed in a 50*50 cm squared arena and the second and third SIT in a circular arena with a diameter of 100 cm. The total duration of the test was 10 min and consisted of two phases: the habituation and the test phase. During the habituation phase, the experimental rat was permitted to freely explore the arena containing two empty wire mesh cages placed on opposite sides of the arena for 5 min. Then, the rat was returned to its home cage and the researcher placed an object and a test rat (age-matched; maximum of 14 days older) in the wire mesh cages. During test phase the experimental rat was put in the arena and allowed to freely explore for 5 min. Behavior was video recorded and analyzed offline using Ethovision XT14 (Noldus Information Technology, Wageningen, The Netherlands). A 10% ethanol solution was used to clean the arena after each test. The time spent with the rat and the time spent with the object were recorded and used as an indicator of social behavior. Rats spending less than 3 s interacting with any of the wire mesh cages were excluded (day 68: 1 RSDadu, day 91: 1 RSDjuv + 1 RSDadu).

### 2.8 PET imaging

PET was used to measure TSPO levels as a marker of microglia reactive to the stressors, on day 46, 74, 89, and 93. Rats were anaesthetized with isoflurane (induction 5% and maintenance 2%, in oxygen) for an intravenous injection of [^11^C]PBR28 in the tail vein. The average injected tracer dose was similar between groups. The average radioactivity dose at the time of injection was 39.6 ± 6.2 MBq. After tracer injection, rats were placed back in their home cage. About 30 min after tracer injection, rats were anaesthetized with isoflurane and positioned into the PET camera (microPET Focus 220, Siemens) for a transmission scan of 10 min followed by an emission scan of 30 min, starting at 45 min after tracer injection. Heating pads and eye salve were used to maintain body temperature and prevent dehydration of the eyes, respectively. Heart rate and blood oxygen levels were monitored. After the scan, rats were placed back into their home cage. After the final scan on day 93, rats were terminated under deep anesthesia by cardiac perfusion.

PET scans were iteratively reconstructed (OSEM2D, 4 iterations, 16 subsets) into a single frame, resulting in images with a 128 × 128 × 95 matrix, a pixel width of 0.632 mm, and a slice thickness of 0.762 mm. PET images were automatically co-registered to a functional [^11^C]PBR28 template [17], which was spatially aligned with a stereotaxic T2-weighted MRI template in Paxinos space [18]. Predefined volumes of interest (VOI) representing specific brain regions were copied to the realigned PET images and used to calculate the tracer uptake, expressed as percentage of the injected dose per gram of tissue. 1 control and 2 RSDjuv rats died before the first PET scan and 1 RSDjuv rat died before the last scan. Due to tracer production failure, 1 RSDadu rat and 2 controls rats could not be scanned on day 68 and day 89, respectively.

### 2.9 Brain collection and immunohistochemistry

To collect brains for immunostaining, rats were perfused with PBS and 4% paraformaldehyde (PFA) under deep anesthesia. Brains were fixed for 48 h in 4% PFA at room temperature. After dehydration in 25% sucrose at 4 °C, the brains were embedded with optimal cutting temperature OCT compound and stored at −80 °C.

For immunohistochemistry, 15 μm sections were prepared by cryosection. After washing three times with PBS, antigen retrieval was performed by pressure cooking for 10 min in 10 mM sodium citrate, pH 6.0. Subsequently, the sections were washed and incubated in PBS with 0.3% hydrogen peroxide (H_2_O_2_) for 30 min to block endogenous peroxidases. The sections were then washed and blocked for 30 min with 2% normal donkey serum (NDS; Jackson Immuno Research, 017-000-121) in PBS with 1% Triton X-100 (PBS^+^) and 2% bovine serum albumin (BSA). The sections were then incubated with the primary rabbit-α-ionized calcium-binding adapter molecule 1 (Iba1) antibody (1:2000; Wako, 01–19,741) with PBS^+^ and 1% BSA overnight at 4 °C. The following day, the sections were washed and incubated with the biotinylated secondary donkey-α-rabbit IgG antibody (1:400; Jackson Immuno Research, 711-065-152) for 2 h. After washing with PBS, the section were incubated with Avidin/Biotinylated enzyme Complex ABC solution (VECTASTAIN® ABC Kit, Vector Laboratories, PK-6100) for 30 min. The sections were washed and stained using 0.04% 3,3′-Diaminobenzidine and 0.03% hydrogen peroxide for 10 min and subsequently dehydrated using a sequence of increasing ethanol concentrations. After being air-dried, the slides were mounted with coverslips using DePex (Serva) and stored at room temperature.

### 2.10 Microglial density and spatial distribution analysis

Microglia densities in the parietal and temporal cortices were determined by counting all Iba1 positive cells in a specified cortical region of interest (ROI) of known dimensions (0.28 mm^2^) at a 20X magnification using ImageJ software (http://rsb.info.nih.gov/ij/). The spatial distribution of microglia in the cortex and the distance of the microglia to their nearest neighbor, that is, the average Euclidian distances between the nearest cells, were determined using the NND plugin for ImageJ. Between 8 and 12 ROIs per animal were selected for the analysis. Five rats per group were used.

### 2.11 Plasma corticosterone with ELISA

PET acquisition was performed in the morning or in the afternoon. We observed no difference in corticosterone between morning and afternoon. At the time of each PET acquisition, about 0.50 mL blood samples were collected from the tail vein, prior to injection of the PET tracer, and immediately centrifuged at 5000 g for 3 min. Plasma was collected, frozen in liquid nitrogen and stored at −80 °C. Plasma corticosterone measurement was performed using an enzyme-linked immunoassay (ELISA) using a commercially available kit (Arbor Assays, DetectX Corticosterone Immunoassay kit) according to the manufacturer's recommendations.

### 2.12 Statistical analysis

Statistical analysis of body weight, behavior, plasma corticosterone levels and PET data was performed using SPSS (IBM SPSS Statistics, Version 22.0). A generalized estimating equation (GEE) analysis was performed, using ‘social defeat’ and ‘time’ as factors for longitudinal data statistical analyses, as this analysis can account for missing data. A paired *t*-test was used to assess differences in RSD severity and within groups in the social interaction test. A one-way ANOVA was performed to assess differences between groups in Iba1 staining. The box represents the interquartile range, the whiskers the min to max values, and the center line indicates the median.

## 3 Results

### 3.1 Juvenile rats were exposed to less severe social defeat than adult rats

Average attack latency (Supplementary figure 1A) was significantly lower in rats exposed to RSD in adulthood than during juvenile age (RSDjuv = 102 ± 112, RSDadu = 23 ± 21, p < 0.001). The number of times the intruder was not submitted (RSDjuv = 88%, RSDadu = 30%, p < 0.001) were significantly different between rats exposed to social defeat during juvenile age and adulthood (Supplementary figure 1B).

### 3.2 Juvenile but not adulthood RSD reduced body weight

**Fig. 2:**
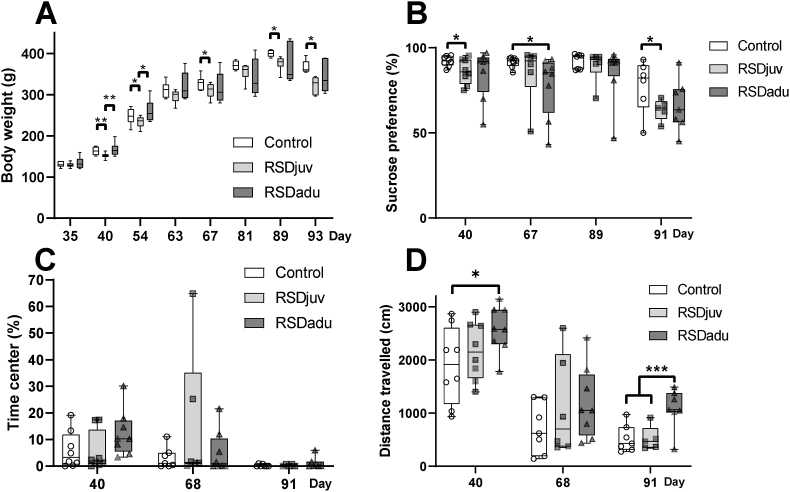
**Bodyweight, anhedonia, locomotion and anxiety changes. A.** Bodyweight (n = 5 to 8 per group). **B.** Anhedonia (n = 5 to 8 per group). **C.** Anxiety-like behavior (n = 5 to 8 per group). **D.** Locomotion (n = 5 to 8 per group). Day 40 is one day after juvenile RSD, day 68 is one day after adulthood RSD, and day 91 is one day after LPS injection. Box represents the interquartile range, the whiskers the min to max values and the center line indicates the median. Statistically significant differences between groups are indicated by asterisks: *p < 0.05, **p < 0.01, ***p < 0.001. Significant differences between time points are not shown.

Body weight of all groups increased with time (p < 0.001, Fig. 2A.). We observed a main effect of RSDjuv (p = 0.003) but not RSDadu (p = 0.634) on body weight. On day 40, the body weight of RSDjuv rats was significantly lower compared to control and RSDadu rats (control: −7%, p = 0.004, RSDadu: −9%, p = 0.010). This difference was still observed on day 54 (control: −6%, p = 0.050, RSDadu: −10%, p = 0.029), whereas only the difference compared to the control group remained statistically significant on day 67 (control: −5%, p = 0.050), and day 89 (control: −6%, p = 0.001). The body weight of RSDadu rats was not significantly different from that of controls at any time point. On day 93, 2 days after LPS injection, the body weight of the RSDjuv group (−12%, p < 0.0001), but not of the RSDadu group (−6%, p = 0.115), was significantly lower than that of controls. Within group comparison between day 89 and day 93, the period in which LPS was injected, showed a reduction in bodyweight in control (−8%, p < 0.0001), RSDjuv (−15%, p < 0.0001), and RSDadu (−8%, p = 0.031) rats.

### 3.3 Juvenile but not adulthood RSD induced a synergistic decrease in sucrose preference following LPS injection

The sucrose preference test was used to assess anhedonia (Fig. 2. B). Overall, we observed a main effect of time (p < 0.001) but not of RSDjuv (p = 0.217) or RSDadu (p = 0.181). On day 40, rats submitted to the RSD protocol at juvenile age showed a reduction in sucrose intake compared to controls (−7%, p = 0.023). On day 67, rats submitted to the RSD protocol during adulthood showed a reduction in sucrose uptake compared to controls (−15%, p = 0.024). On day 91, following the LPS injection, RSDjuv rats, but not RSDadu rats, showed a significant reduction in sucrose intake compared to controls (RSDjuv: −18%, p = 0.027, RSDadu: −15% p = 0.664). Within-group comparison showed a consistent decrease in sucrose preference between day 89 and day 91 (period during which LPS was injected) in the control (−16%, p = 0.018), RSDjuv (−29%, p < 0.0001) and RSDadu (−23%, p = 0.003) group.

### 3.4 RSD did not significantly affect anxiety-like behavior nor locomotion

The percentage of time spent in the center of the arena and the total distance travelled in the OFT were used to assess anxiety-like behavior (Fig. 2C.) and locomotion (Fig. 2D.), respectively. We observed a main effect of time (p < 0.001) and RSDadu (p = 0 < 001) but not of RSDjuv (p = 0.271) on locomotion. RSDadu travelled significantly more than controls on day 40 (p < 0.001) and day 91 (p < 0.0001). There was no main effect in the percentage of time the animal spent in the center or any significant difference between groups at any timepoint.

### 3.5 Juvenile but not adulthood RSD altered social preference following LPS injection

**Fig. 3:**
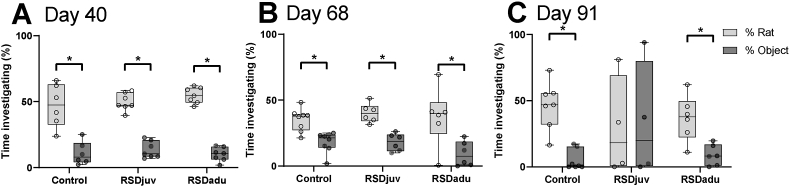
**Social behavioral changes.** Social preference on **A.** Day 40 (following juvenile RSD), **B.** Day 68 (following adulthood RSD during), **C.** Day 91 (following LPS injection). N = 4 to 8 per group. Box represents the interquartile range, the whiskers the min to max values and the center line indicates the median. Statistically significant differences between the time spent interacting with the rat and the object are indicated by asterisks: *p < 0.05. Significant differences between time points are not shown.

To test social behavior, we quantified the preference of a rat for interacting with another rat versus an object. During the familiarization phase, we observed no difference in time spent with the empty wire mesh cages and in distance travelled between groups (data not shown). There was no significant difference in percentage of time spent with the rat between groups at any timepoints (data not shown). On day 40 (p < 0.001) and day 68 (p < 0.01), all groups showed social preference for a rat over an object (Fig. 3. A. B.). Unlike control (p < 0.001) and RSDadu (p < 0.01) rats, RSDjuv (p = 0.89) rats did not show social preference for a rat over an object anymore, one day after the exposure to LPS (day 91) (Fig. 3C.).

### 3.6 RSD increased corticosterone levels in adulthood

**Fig. 4:**
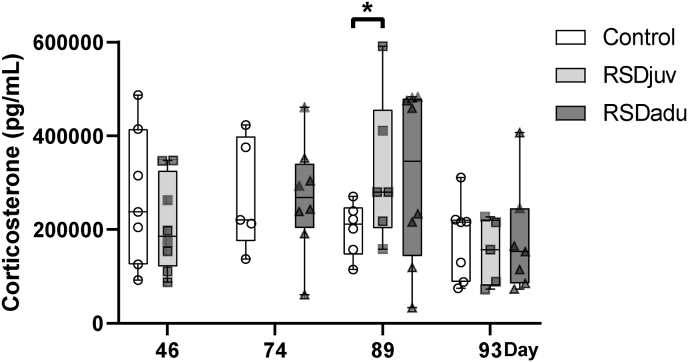
**Plasma corticosterone levels.** n = 5 to 8 rats per group. Day 46 is 6 days after juvenile RSD, day 74 is 6 days after adulthood RSD, day 89 precedes LPS injection, day 93 is 3 days after LPS injection. Box represents the interquartile range, the whiskers the min to max values and the center line indicates the median. Statistically significant differences between groups are indicated by asterisks: *p < 0.05. Significant differences between time points are not shown.

Serum concentrations of corticosterone were measured 6 days after RSD and 1 day before and 3 days after LPS injection (Fig. 4.). Six days after exposure to RSD during juvenile age (day 46) or adulthood (day 74), the corticosterone levels were not different from controls. On day 89, the corticosterone levels were significantly higher in RSDjuv (+61%, p = 0.048), when compared to controls. In RSDadu rats, the corticosterone levels were also higher than in controls, but this was not statistically significant (+56%, p = 0.083). We observed a main effect of time on corticosterone (p < 0.050). On day 91, following the LPS injection, corticosterone levels were not different anymore between groups. Within-group comparison between day 89 and day 93, the period during which LPS was injected, showed that serum corticosterone levels were significantly reduced in the RSDjuv group (−53%, p = 0.002). A reduction was also observed in the RSDadu group (−43%, p = 0.068), but this was not statistically significant. No change was observed for the control group (−11%, p = 0.169).

### 3.7 RSD induced a long-lasting increase in reactive microglia, synergistically enhancing the effect of LPS injection

**Fig. 5:**
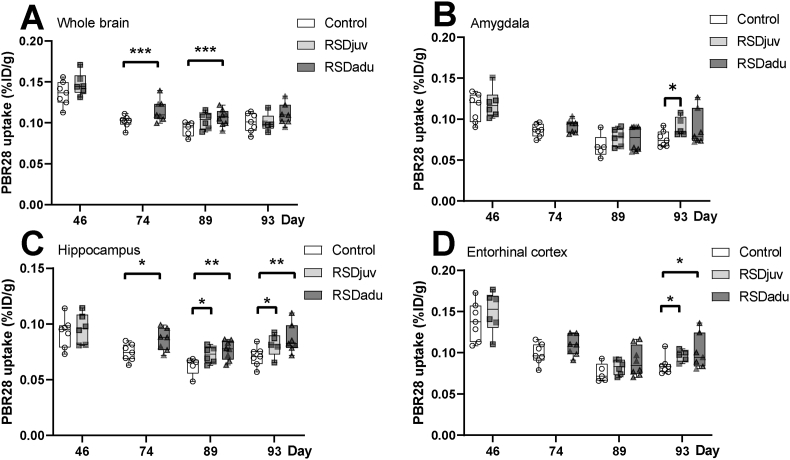
**Microglial reactivity in the whole brain (A.), amygdala (B.), hippocampus (C.) and entorhinal cortex (D.**), as measured with [^11^C]PBR28 PET (n = 5 to 8 per group). Day 46 is 6 days after juvenile RSD, day 74 is 6 days after adulthood RSD, day 89 precedes LPS injection, day 93 is 3 days after LPS injection. Box represents the interquartile range, the whiskers the min to max values and the center line indicates the median. Statistically significant differences between groups are indicated by asterisks: *p < 0.05, **p < 0.01, ***p < 0.001. Significant differences between time points are not shown.

To determine the effect of RSD and LPS on glial activity, [^11^C]PBR28 PET was performed on day 46, 74, 89, and 93 (Fig. 5A. B.C.D.; Table 1).

**Table 1:** **[**^**11**^**C]PBR28 PET: tracer uptake in the brain** of control animals (control), and animals exposed to social defeat at juvenile age (RSD-juv) or adulthood (RSD-adu). Tracer uptake (% injected dose/g) is presented for different brain areas. Data are shown as mean ± SD. Statistically significant differences compared to control animals on the same day are indicated with an asterisk: *p < 0.05; **p < 0.01, ***p < 0.001. Statistically significant differences between RSDjuv and RSDadu on the same day are indicated with a ^$^: ^$^p < 0.05; ^$$^p < 0.01, ^$$$^p < 0.001.

Brain regions	Day 46 (after RSDjuv)		Day 74 (after RSDadu)		Day 89 (before LPS)			Day 93 (after LPS)		
	Control	RSDjuv	Control	RSDadu	Control	RSDjuv	RSDadu	Control	RSDjuv	RSDadu
Amygdala	0.11 ± 0.017	0.12 ± 0.018	0.086 ± 0.008	0.092 ± 0.008	0.067 ± 0.014	0.078 ± 0.011	0.077 ± 0.015	0.076 ± 0.010	0.091 ± 0.012*	0.089 ± 0.022
BNST	0.084 ± 0.018	0.087 ± 0.027	0.073 ± 0.012	0.083 ± 0.011	0.059 ± 0.017	0.078 ± 0.009*	0.071 ± 0.011	0.079 ± 0.015	0.074 ± 0.015	0.076 ± 0.007
Basal Ganglia	0.087 ± 0.010	0.082 ± 0.020	0.067 ± 0.007	0.071 ± 0.012	0.058 ± 0.008	0.065 ± 0.010	0.063 ± 0.008	0.064 ± 0.009	0.074 ± 0.004**	0.071 ± 0.018
Brainstem	0.123 ± 0.013	0.123 ± 0.012	0.102 ± 0.009	0.105 ± 0.015	0.083 ± 0.010	0.092 ± 0.006	0.101 ± 0.015*	0.094 ± 0.009	0.104 ± 0.015	0.110 ± 0.017*
Cerebellum	0.173 ± 0.022	0.174 ± 0.024	0.133 ± 0.017	0.161 ± 0.027*	0.116 ± 0.013	0.137 ± 0.014**	0.159 ± 0.019***^$$^	0.133 ± 0.015	0.131 ± 0.014	0.157 ± 0.012***^$$$^
Corpus callosum	0.094 ± 0.012	0.102 ± 0.016	0.076 ± 0.005	0.091 ± 0.013**	0.072 ± 0.010	0.082 ± 0.015	0.086 ± 0.009**	0.081 ± 0.013	0.078 ± 0.013	0.089 ± 0.012
Cortex Entorhinal	0.138 ± 0.023	0.149 ± 0.025	0.099 ± 0.013	0.109 ± 0.013	0.076 ± 0.011	0.082 ± 0.009	0.090 ± 0.018	0.085 ± 0.011	0.096 ± 0.007*	0.101 ± 0.021*
Cortex Frontal	0.195 ± 0.021	0.230 ± 0.019**	0.130 ± 0.012	0.151 ± 0.024*	0.129 ± 0.017	0.137 ± 0.017	0.131 ± 0.013	0.135 ± 0.021	0.120 ± 0.022	0.135 ± 0.014
Cortex Frontal Association	0.310 ± 0.032	0.380 ± 0.034**	0.200 ± 0.026	0.220 ± 0.041	0.175 ± 0.018	0.205 ± 0.024**	0.192 ± 0.030	0.194 ± 0.021	0.178 ± 0.029	0.205 ± 0.027
Cortex Insular	0.166 ± 0.023	0.177 ± 0.018	0.099 ± 0.007	0.110 ± 0.015	0.082 ± 0.009	0.088 ± 0.011	0.088 ± 0.016	0.087 ± 0.011	0.099 ± 0.006**	0.100 ± 0.022
Cortex Medial Prefrontal	0.154 ± 0.023	0.166 ± 0.023	0.104 ± 0.013	0.125 ± 0.034	0.094 ± 0.018	0.098 ± 0.015	0.104 ± 0.019	0.108 ± 0.013	0.096 ± 0.021	0.107 ± 0.023
Cortex Occipital	0.136 ± 0.012	0.165 ± 0.013***	0.111 ± 0.011	0.134 ± 0.019**	0.117 ± 0.022	0.130 ± 0.026	0.135 ± 0.013	0.120 ± 0.023	0.100 ± 0.018	**0.125 ± 0.013$$**
Cortex Orbitofrontal	0.220 ± 0.033	0.240 ± 0.028	0.126 ± 0.019	0.145 ± 0.026	0.121 ± 0.018	0.130 ± 0.013	0.126 ± 0.025	0.120 ± 0.014	0.122 ± 0.013	0.137 ± 0.022
Cortex Parietal	0.149 ± 0.017	0.175 ± 0.014**	0.110 ± 0.006	0.129 ± 0.016**	0.117 ± 0.020	0.123 ± 0.021	0.120 ± 0.010	0.117 ± 0.021	0.101 ± 0.019	0.113 ± 0.008
Cortex Temporal	0.158 ± 0.022	0.171 ± 0.021	0.106 ± 0.008	0.115 ± 0.013	0.086 ± 0.007	0.094 ± 0.008*	0.098 ± 0.017	0.091 ± 0.012	0.103 ± 0.008*	0.108 ± 0.020*
Forebrain	0.099 ± 0.017	0.105 ± 0.019	0.081 ± 0.010	0.085 ± 0.012	0.072 ± 0.012	0.080 ± 0.010	0.078 ± 0.013	0.082 ± 0.012	0.086 ± 0.012	0.087 ± 0.015
Hippocampus	0.093 ± 0.013	0.096 ± 0.013	0.074 ± 0.008	0.086 ± 0.010*	0.063 ± 0.008	0.072 ± 0.007*	0.077 ± 0.009**	0.071 ± 0.009	0.081 ± 0.010*	0.087 ± 0.013**
Midbrain	0.098 ± 0.011	0.087 ± 0.016	0.089 ± 0.010	0.090 ± 0.013	0.078 ± 0.011	0.080 ± 0.006	0.090 ± 0.012*^$^	0.094 ± 0.015	0.094 ± 0.011	0.102 ± 0.017
Nucleus Accumbens	0.120 ± 0.015	0.120 ± 0.029	0.075 ± 0.010	0.096 ± 0.021*	0.062 ± 0.015	0.075 ± 0.009	0.079 ± 0.022	0.077 ± 0.013	0.090 ± 0.007**	0.092 ± 0.029
Striatum	0.086 ± 0.011	0.086 ± 0.019	0.067 ± 0.007	0.076 ± 0.017	0.056 ± 0.005	0.068 ± 0.008**	0.066 ± 0.011**	0.066 ± 0.010	0.071 ± 0.007	0.075 ± 0.016
Thalamus	0.101 ± 0.019	0.106 ± 0.019	0.083 ± 0.010	0.085 ± 0.012	0.075 ± 0.013	0.082 ± 0.011	0.079 ± 0.013	0.083 ± 0.012	0.088 ± 0.013	0.088 ± 0.015
Whole Brain	0.136 ± 0.015	0.147 ± 0.011	0.102 ± 0.007	0.115 ± 0.014*	0.092 ± 0.009	0.103 ± 0.010	0.106 ± 0.010*	0.101 ± 0.012	0.100 ± 0.011	0.110 ± 0.013

A main effect of time and group was observed for the whole brain tracer uptake (p < 0.0001). RSD induced a significant increase in tracer uptake in RSDjuv rats on day 46 in the frontal (+18%, p < 0.001), frontal association (+23%, p < 0.001), occipital (+21%, p < 0.0001), and parietal cortices (+18%, p < 0.001). In RSDadu rats, significantly enhanced tracer uptake in the whole brain (+13%, p = 0.011), cerebellum (+21%, p = 0.012), corpus callosum (+20%, p < 0.01), nucleus accumbens (+28%, p = 0.01), hippocampus (+16%, p = 0.011) and frontal (+16%, p = 0.026), parietal (+17%, p < 0.01) and occipital cortices (+21%, p < 0.01) was observed on day 74, when compared to control animals. RSD also induced a long-lasting significant increase in tracer uptake in the frontal association (+17%, p < 0.01) and temporal cortices (+9%, p = 0.050), bed nucleus of the stria terminalis (BNST) (+32%, p = 0.013), cerebellum (+18%, p < 0.01), striatum (+21%, p < 0.001), and hippocampus (+14%, p = 0.021) of RSDjuv rats on day 89 (before LPS injection), and in the whole brain (+15%, p < 0.01), cerebellum (+37%, p < 0.01), corpus callosum (+19%, p < 0.01), striatum (+18%, p = 0.001), hippocampus (+22%, p = 0.001), midbrain (+15%, p = 0.042), and brainstem (+22%, p < 0.01) of RSDadu rats, when compared to control animals. On day 89, RSDadu rats had a significantly higher tracer uptake in the midbrain (+13%, p = 0.042) and cerebellum (+16%, p = 0.01) than RSDjuv rats.

Three days after LPS injection (day 93), tracer uptake was significantly higher in the entorhinal (+13%, p = 0.017), insular (+14%, p < 0.01), and temporal cortices (+13%, p = 0.023), amygdala (+20%, p = 0.010), nucleus accumbens (+17%, p < 0.01), hippocampus (+14%, p = 0.042), and basal ganglia (+16%, p < 0.01) of RSDjuv rats, and in the entorhinal (+19%, p = 0.047), and temporal cortices (+19%, p = 0.037), cerebellum (+18%, p = 0.0001), hippocampus (+23%, p < 0.01), and brainstem (+17%, p = 0.019) of RSDadu rats, when compared to control animals. On day 93, RSDadu rats had a significantly higher tracer uptake in the occipital cortex (+25%, p < 0.01) and cerebellum (+20%, p = 0.0001) than RSDjuv rats.

Within group comparison between day 89 and day 93, the period during which LPS was injected, showed that tracer uptake in control rats had significantly increased in the BNST (+34%, p = 0.026), cerebellum (+15%, p = 0.021), frontal association (+11%, p = 0.007), and medial prefrontal cortices (+15%, p = 0.050), striatum (+18%, p = 0.016), midbrain (+21%, p = 0.020), brainstem (+13%, p = 0.45). RSDjuv rats showed a significant increase in tracer uptake in the amygdala (+17%, p = 0.001), entorhinal (+17%, p < 0.001), insular (+13%, p < 0.001), and temporal cortices (+10%, p = 0.023), midbrain (+18%, p = 0.003), brainstem (+13%, p = 0.44), and basal ganglia (+14%, p < 0.0001), but a significantly lower tracer uptake in the frontal association (−13%, p = 0.008) and frontal cortices (−12%, p = 0.018) over this period. RSDadu rats had a significantly higher tracer uptake in the entorhinal (+12%, p = 0.019), insular (+14%, p = 0.11), and orbitofrontal cortices (+9%, p = 0.003), striatum (+14%, p = 0.038), hippocampus (+13%, p = 0.001), thalamus (+11%, p = 0.010), midbrain (+13%, p = 0.021), brainstem (+9%, p = 0.26) and forebrain (+12%, p = 0.008) on day 93 than on day 89.

### 3.8 Previous exposure to RSD synergistically increased microglial density following LPS exposure at adulthood

**Fig. 6:**
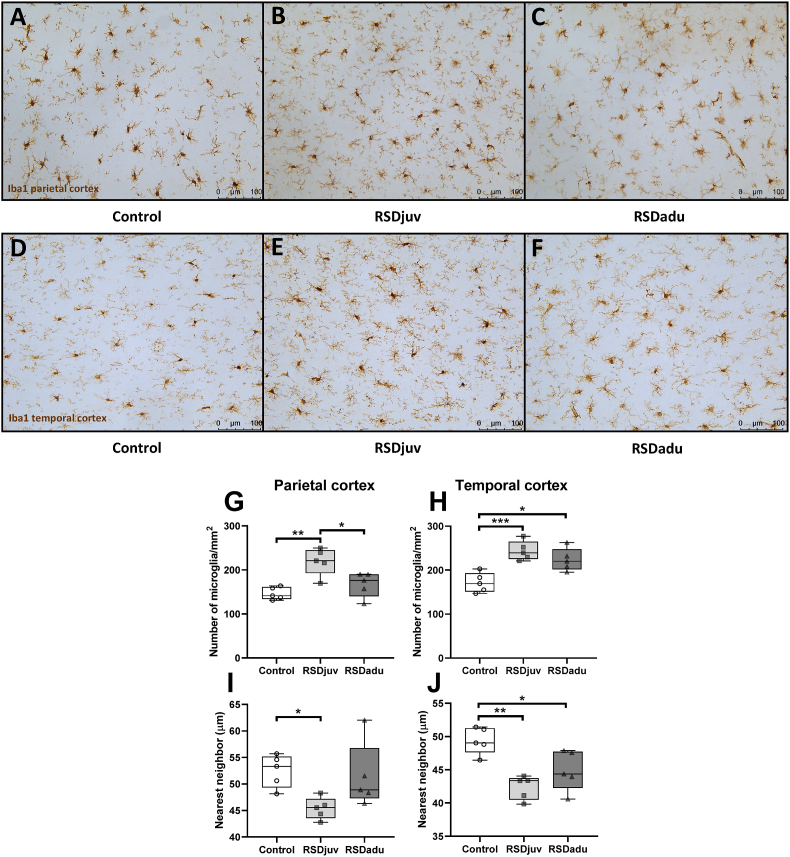
**Density of microglia 3 days after the LPS injection on day 93.** Representative Iba1 staining of microglia in the parietal cortex of control (**A.**), RSDjuv (**B.**), and RSDadu (**C.**) rats and temporal cortex of control (**D.**), RSDjuv (**E.**), and RSDadu (**F.**) rats. Number of Iba1-positive cells/mm2 in the parietal (**G.**) and temporal (**H.**) cortices. Nearest neighbor distance analysis in the parietal (**I.**) and temporal (**J.**) cortices. n = 5 rats per group. Box represents the interquartile range, the whiskers the min to max values and the center line indicates the median. Statistically significant differences between groups are indicated by asterisks: *p < 0.05, **p < 0.01, ***p < 0.001.

To determine the effect of RSD and LPS on microglia, the effect on microglia cell density was determined in the parietal (Fig. 6 A.B.C.) and temporal (Fig. 6 D.E.F.) cortices by counting the number of Iba1-positive cells.

On day 93, 3 days after the injection of LPS, the density of microglia cells in the parietal (+50%, p < 0.01) and temporal cortices (+43%, p < 0.001) was significantly higher in the rats previously exposed to juvenile RSD than in control rats injected with LPS (Fig. 6G. H.). The density of microglia cells in the parietal cortex (+31%, p < 0.05) of rats previously exposed to RSD during juvenile age was also significantly higher than in rats previously exposed to RSD at adulthood. Rats previously exposed to RSD at adulthood had a higher microglial density in the temporal (+31%, p < 0.05) but not parietal cortex, when compared to control rats injected with LPS.

In line with the increased microglial density, a significant decrease in the nearest neighbor distance between microglia in the parietal (−16%, p < 0.05) and temporal (−14%, p < 0.01) cortices of rats exposed to juvenile RSD and LPS was observed, as compared to control rats injected with LPS (Fig. 6. I.J.). Rats previously exposed to RSD at adulthood also had a significant decrease in the nearest neighbor distance between microglia in the temporal (−10%, p < 0.05), but not parietal cortex, compared to control rats exposed to LPS only.

## 4 Discussion

The present findings indicate that exposure to RSD at juvenile age or adulthood induced similar short-term anhedonia-like behavior, a long-lasting increase in plasma corticosterone and microglial reactivity, but no change in anxiety or social behavior. In addition, iba1 staining and within-group TSPO PET imaging revealed that exposure to RSD synergistically increased microglia cell density and reactive microglia following an immune challenge at adulthood. This effect was less pronounced in rats previously exposed to RSD during adulthood. Rats exposed to juvenile RSD in combination with an immune challenge at adulthood exhibited enhanced anhedonia and social interaction dysfunction. Such effects were not observed when RSD occurred during adulthood, indicating that the susceptibility to RSD is age dependent.

RSD is a validated model of social stress that mimics some of the effects of bullying in humans (Björkqvist, 2001). In accordance with other studies (Iñiguez et al., 2014; Kopschina Feltes et al., 2017), we found that juvenile and adult rats exposed to five days of social defeat developed short, but not long term, anhedonia-like behavior as indicated by a reduced sucrose preference in the SPT. However, our RSD model did not induce anxiety-like behavior, altered locomotion, or social dysfunction, as the time spent in the center of the open field, the distance travelled, and the social interaction were not affected. Previous studies using the RSD protocol during juvenile age or adulthood have shown contradictory results, as either an increase in anxiety and social dysfunction (MacKay et al., 2017; Mancha-Gutiérrez et al., 2021; Mancini et al., 2021), or no effect (Moraga-Amaro et al., 2022; Mouri et al., 2018) have been reported. Many factors may explain such differences, including housing conditions, differences in the RSD procedure, its duration and timing, and the interval between the RSD and the behavioral tests.

Infection by injection of LPS is a well-validated model to challenge the immune system. In the present study, LPS injected in adulthood (day 90) induced sickness behavior and reduced body weight and sucrose preference in SPT. This is consistent with findings in animal studies demonstrating sickness behavior following LPS injection (Bassi et al., 2012) and clinical data indicating similar behavior in response to an LPS injection or an infection (Fullerton et al., 2016). The observed LPS effects on behavior were more pronounced when rats were previously exposed to RSD during juvenile age. Sucrose preference dropped significantly more in the rats exposed to juvenile RSD. In addition, only the combination of RSD and LPS induced dysfunction of social interaction characterized by a lack of social preference towards a rat and an object, whereas RDS or LPS alone had no effect. These results indicate that social stress experienced during juvenile age affects the response to immune challenges in adulthood.

The synergistic effect of an immune challenge during adulthood and RSD during juvenile age, but not during adulthood, is an interesting observation as resident rats were less aggressive towards juvenile than adult intruder rats. Residents took more time to attack and spent less time attacking juvenile rats than adult intruder rats. Furthermore, juvenile intruder rats showed less avoidance behavior and were submitted less often than adult intruder rats. Therefore, the level of stress experienced by the juvenile rats during the social encounter is expected to be weaker than that experienced by adult rats. Another study made similar observations in mice (Montagud-Romero et al., 2017). However, despite this apparent weaker threat, juvenile social defeat has induced long term consequences, characterized by a reduction in body weight, higher plasma corticosterone levels in adulthood and enhanced susceptibility to an immune challenge, which were not observed if the social defeat occurred during adulthood. These results suggest that the timing of the insult is important, and that juvenile age is a period of higher susceptibility to stressors than adulthood. During juvenile age many neurodevelopmental changes still take place, such as neurogenesis, myelination, and synaptic pruning, which could explain increased vulnerability to stress at juvenile age (Hefner and Holmes, 2007; Spear, 2013; Yoo et al., 2013).

In the present work, we found that juvenile RSD increased corticosterone levels in the plasma in adulthood relative to control animals. This increase in plasma corticosterone levels suggests an altered activity of the hypothalamus-pituitary (HPA) axis, believed to be an indicator of heightened stress sensitivity (Holtzman et al., 2013). Such heightened stress sensitivity characterized by higher corticosterone levels, is common in individuals exposed to childhood adversity and at risk for psychosis and is an important psychological process in the development of psychotic experiences in the early stages of schizophrenia (Reininghaus et al., 2016; Walder et al., 2000). It has been observed that juvenile social stress in mice increased corticosterone serum levels in juvenile age and adulthood and induced long term memory impairments (Mancha-Gutiérrez et al., 2021). Conversely, another study observed reduced corticosterone levels in adulthood in rats exposed to juvenile social stress (Mancini et al., 2021). Interestingly, they also observed that juvenile social stress confers resilience against emotional behavior and spatial memory dysfunction induced by exposure to stressors later in life. Many factors may explain such differences with their and our study, including the rat species used, the timing of the RSD protocol as they performed it between day 28 and 34 while we did it between day 35 and 40, differences in RSD procedure, and in the adulthood stressor as they performed a single prolonged stress (2 h restraint stress followed by 15min forced swim and isoflurane until loss of consciousness) while we conducted an immune challenge by injecting LPS (Mancini et al., 2021).

In our study, enhanced glial reactivity to the LPS injection was apparent in several brain regions after the combination of social stress and the LPS immune challenge, as compared to only an LPS challenge. Non-invasive [^11^C]PBR28 PET imaging revealed that LPS injection alone increased TSPO levels, a protein associated with microglial reactivity in several brain regions, such as the frontal cortex, striatum, and brainstem. In accordance with other studies (Moraga-Amaro et al., 2022; Rodríguez-Arias et al., 2018), *s*ocial stress during juvenile age or adulthood induced a long-lasting increase in reactive microglia in brain regions related to stress-induced psychopathologies, such as the frontal cortex, striatum, brainstem and hippocampus. This result is in line with human studies that observed a link between early life stress characterized as childhood maltreatment and being raised in a ‘harsh family’ and heightened peripheral and central markers of inflammation (Danese and J Lewis, 2017; Miller and Chen, 2010). Furthermore, our TSPO PET results have translational value as TSPO tracers are also used clinically and imaging studies observed increased proinflammatory states and TSPO upregulation in the hippocampus, amygdala, prefrontal cortex, and the anterior cingulate cortex in people suffering from major depressive disorder (Gritti et al., 2021; Kim and Won, 2017).

The longitudinal within-group comparisons using in vivo TSPO PET imaging revealed that the increased microglial reactivity to the LPS challenge was more pronounced when the rats were previously exposed to social adversity during juvenile age or adulthood. The microglial reactivity as indicated by TSPO upregulation was significantly higher in the basal ganglia, amygdala, nucleus accumbens, temporal, entorhinal and insular cortices of the rats previously exposed to juvenile RSD. Microglial cell density, as measured with iba1 staining, was higher in the temporal and parietal cortices of rats previously exposed to juvenile RSD. This effect was less pronounced in rats previously exposed to adulthood RSD. These results indicate the increased susceptibility induced by the previous exposure to social stress may alter the responsivity of the immune system. Another research group observed that previous exposure to social adversity enhanced the peripheral and central inflammatory response to a subsequent acute social stress challenge one week later (Finnell et al., 2019). Furthermore, our results are also in accordance with a similar study that observed that RSD enhanced the microglial response in the hippocampus, ventricular nucleus and PFC following an innate immune challenge consisting in injecting LPS 4 h after the last episode of social adversity (Wohleb et al., 2012). Our studies are complementary as they observed short-term priming of the immune system following the last episode of social stress, while we observed that this priming effect remained for more than a month following the last stressor.

Long-lasting microglial reactivity to the stressors was apparent in several brain regions important for the regulations of emotions, stress, memory, and cognition and known to be involved in psychopathologies, such as schizophrenia. The frontal association cortex and hippocampus are primary integrators of the stress response (McEwen et al., 2016). These regions keep developing and maturing throughout that juvenile age and until early adulthood (Andersen and Teicher, 2008; He and Crews, 2007). Increased inflammation in these regions may compromise their development and underlie the long-term behavioral alterations induced by social stress and increased the susceptibility to stressors and immune challenges later in life. For example, extended activation of CD11b + cells in the hippocampus is associated with decreased neurogenesis and cognitive impairment (Wohleb et al., 2012).

Despite that the use of in vivo PET imaging proved useful to non-invasively monitor the effects of social stress and LPS on microglial states in vivo, our study includes several limitations. Limitations to this study include the absence of direct measurement of brain and plasma pro- and anti-inflammatory cytokines such as interleukin 1 b, and interleukin 10, respectively. Furthermore, our study design does not allow to find causality between the changes in corticosterone levels, microglia, and behavior. Future studies using drugs such as colony-stimulating factor 1 receptor inhibitors to deplete microglia during the stressors could verify causality.

## 5 Conclusion

Our results indicate that previous exposure to social adversity increased sensitivity to an immune challenge later in life. The stress-induced enhanced sensitivity was accompanied by a long-term increase in plasma corticosterone levels and microglial reactivity to the RSD. However, only the rats exposed to the social stress during juvenile age, but not adulthood, exhibited increased microglial density in the frontal cortex and enhanced susceptibility towards the development of anhedonia and social interaction dysfunction after the immune challenge. This suggests that juvenile social stress can have more deleterious effects in the long term than a similar stress in adulthood.

## Formatting of funding sources

This research did not receive any specific grant funding agencies in the public, commercial, or not-for-profit sectors.

## CRediT authorship contribution statement

**Cyprien G.J. Guerrin:** Conceptualization, Methodology, Formal analysis, Investigation, Writing – original draft. **Janine Doorduin:** Conceptualization, Methodology, Writing – review & editing, Supervision. **Kavya Prasad:** Investigation, Writing – review & editing. **Daniel A. Vazquez-Matias:** Investigation, Writing – review & editing. **Lara Barazzuol:** Resources, Writing – review & editing. **Erik F.J. de Vries:** Conceptualization, Methodology, Writing – review & editing, Supervision.

## Declaration of competing interest

The authors declare that they have no known competing financial interests or personal relationships that could have appeared to influence the work reported in this paper.
