# Supplementary figures and images for: Social adversity during juvenile age but not adulthood increases susceptibility to an immune challenge later in life
Source: Neurobiol Stress. 2023 Feb 8;23:100526. doi: 10.1016/j.ynstr.2023.100526 (PMC9945751; doi:10.1016/j.ynstr.2023.100526)

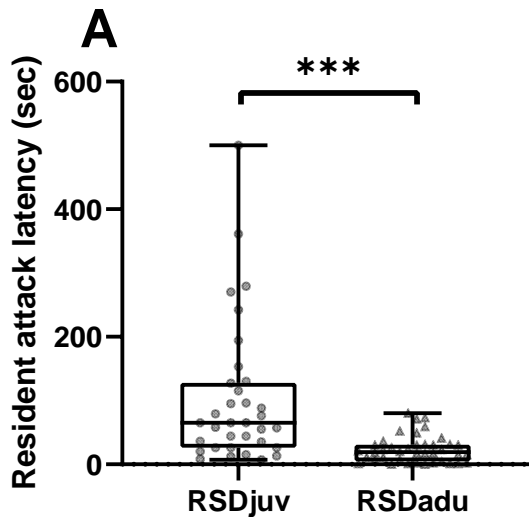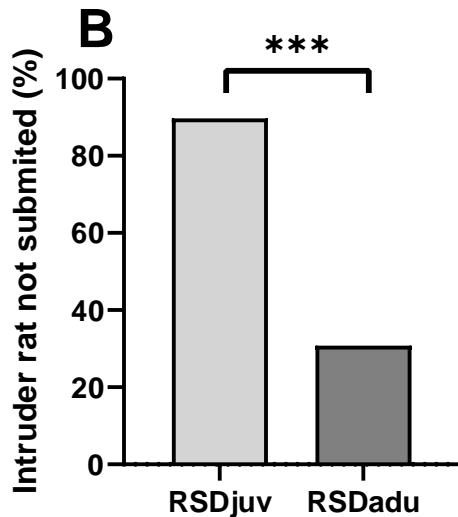

Supplement: Supplementary file 1 [file mmc1.pdf]
